# A Federated Attention-Based Multimodal Biometric Recognition Approach in IoT

**DOI:** 10.3390/s23136006

**Published:** 2023-06-28

**Authors:** Leyu Lin, Yue Zhao, Jintao Meng, Qi Zhao

**Affiliations:** Science and Technology on Communication Security Laboratory, Chengdu 610041, China; ilinleyu@outlook.com (L.L.); mengjintao01@126.com (J.M.); zq8484@yeah.net (Q.Z.)

**Keywords:** federated learning, multimodal system, person recognition, attention mechanism, IoT

## Abstract

The rise of artificial intelligence applications has led to a surge in Internet of Things (IoT) research. Biometric recognition methods are extensively used in IoT access control due to their convenience. To address the limitations of unimodal biometric recognition systems, we propose an attention-based multimodal biometric recognition (AMBR) network that incorporates attention mechanisms to extract biometric features and fuse the modalities effectively. Additionally, to overcome issues of data privacy and regulation associated with collecting training data in IoT systems, we utilize Federated Learning (FL) to train our model This collaborative machine-learning approach enables data parties to train models while preserving data privacy. Our proposed approach achieves 0.68%, 0.47%, and 0.80% Equal Error Rate (EER) on the three VoxCeleb1 official trial lists, performs favorably against the current methods, and the experimental results in FL settings illustrate the potential of AMBR with an FL approach in the multimodal biometric recognition scenario.

## 1. Introduction

The Internet of Things (IoT) has gained significant popularity lately, partly because of the increasing prevalence of high-speed networks and smart devices. To ensure security, authentication is the foremost requirement for each user in IoT systems. Although password or key-based identification methods have matured into effective means, biometric features hold a unique position in the field. Unlike keys or ID cards, biometric features cannot be lost, and unlike knowledge-based features such as PINs or security questions, they cannot be forgotten. Therefore, biometric-based security systems can be applied in many IoT fields, such as using biometric locks in smart lock systems for doors, requiring biometric recognition for healthcare providers before prescribing medication in IoT medical systems [1], implementing access control, and monitoring systems.

Current research on biometric recognition has primarily focused on unimodal approaches, which involve utilizing a range of biometric characteristics, including voice, face, gait, and fingerprint [2]. However, unimodal biometric recognition often faces challenges in obtaining accurate feature patterns, resulting in a decline in recognition performance and leaving potential vulnerabilities for attacks. Even previously considered secure method such as vein recognition, has shown weaknesses when subjected to attacks, as the structures of the hand vein can be detected from several meters away under certain circumstances [3]. To address these issues, researchers have explored the benefits of combining multiple modalities by embedding them into a deep feature space [4,5]. The combination of biometric features has shown a positive impact on biometric recognition tasks, an ideal multimodal biometric recognition system can integrate diverse modalities and provide more emphasis to the modality that has better distinguishing features. Moreover, the fusion weights can withstand different factors, including audio-visual backgrounds, and account for incomplete or damaged modalities, leading to a more accurate and robust system. In this paper, we choose the voice and face to achieve multimodal biometric recognition.

The model performance is greatly influenced by the quantity of training data, the training of multimodal biometric recognition models necessitates access to copious amounts of biometric data. In addition, traditional multimodal data fusion tasks face challenges in acquiring significant amounts of data due to the sensitive and proprietary nature of industrial data, as well as concerns regarding user privacy protection. The limited sharing of data creates impediments to realizing the full potential of heterogeneous data in the IoT. Additionally, centralized data processing presents risks of data leakage in practical applications. To address these challenges, Google [6] first proposed the concept of Federated Learning (FL), which trains models separately on various edge devices using their respective training samples, then subsequently aggregates model parameters, therefore facilitating global information sharing without compromising user privacy. The research of a privacy-preserving solution for multimodal biometric recognition can be highly beneficial. FL is an effective mechanism that enables IoT devices to collaboratively train high-quality models while preserving the confidentiality of the training data.

The major contributions of our work are listed as follows:

- We present a novel multimodal biometric recognition model, AMBR. By fusing the face and voice features, our AMBR model collects and condenses the crucial inputs from each modality and shares them with another modality, achieving better performance for person recognition.
- Novel feature extraction approaches with attention mechanisms are developed for each modality. It not only improves the unimodal recognition accuracy but also effectively extracts features from different modalities for the multimodal fusion stage.
- Trained with FL, our model addresses the issue of data interoperability when collecting biometric data from different edge devices, and promotes data communication and collaboration while ensuring higher levels of privacy in the IoT.

The rest of this paper is organized as follows. Section 2 briefly discusses prior research conducted on multimodal biometric recognition and the FL method. Section 3 explains our proposed model. The experimental setup and results are presented in Section 4. Finally, the conclusions of our research are provided in Section 5.

## 2. Related Works

### 2.1. Multimodal Person Recognition

Rapid advancements in artificial intelligence and IoT technologies have emphasized the need for precise user identification methods. Although significant progress has been made in unimodal biometric authentication, such as speaker recognition and face recognition, their performance degrades significantly under more challenging conditions. Recording devices, distance, and background noise can affect the quality of sound information, whereas illumination, pose, distance, and other factors can substantially impact face recognition performance. To address the limitations of unimodal authentication methods, researchers have proposed multimodal biometric recognition technology that expands the application scope of biometric recognition by fusing different types of features, therefore improving the accuracy and anti-attack capability of recognition systems.

Multimodal biometric fusion is a crucial research topic in biometric recognition studies, when utilizing multimodal features for recognition, combining them can alleviate feature overlap and enhance recognition accuracy. Generally, multimodal identity recognition systems demonstrate notable advantages in terms of high reliability, robustness, and broad applicability [7]. Table 1 provides a comparison of the available techniques. Merging data from different modalities is a key issue to be addressed in modeling, after extracting features from different modalities, we integrate the information extracted from various modalities into a stable representation, and the quality of this representation often determines the effect of the final fusion model. Simple concatenation fusion merges different modal data into the same vector space and fuses all modality data through vector concatenation. Inspired by Shons et al. [4], we employ attention-based fusion instead of simple concatenation fusion to fully utilize information gain between modalities, compensate for the shortcomings of simple concatenation fusion, and extract significant features from voice and facial information.

### 2.2. Attention Mechanism

The attention mechanism plays a crucial role in determining where to focus and aids in adaptive feature refinement. Originally employed in neural machine translation with encouraging outcomes, the attention mechanism has now gained significant traction in various computer vision applications. Notably, it has been successfully implemented in tasks such as natural scene text detection [13] and semantic segmentation [14], demonstrating its wide applicability and effectiveness in various domains.

Liu et al. [15] incorporated channel attention into MobileFaceNet to enhance model performance, and Tan et al. [16] proposed three distinct attention mechanisms for recognizing pedestrian attributes, each designed to approach the problem from a unique perspective and access pertinent information. Fu et al. [17] append the attention module to enable the adaptive integration of local features with their global dependencies, resulting in improved segmentation performance on three challenging scene segmentation datasets. Most of these approaches utilize single implementations of attention, which may not be adequate in achieving optimal performance. Furthermore, few studies have investigated the application of attention mechanisms for multimodal recognition tasks. To overcome these limitations, our proposed approach combines multiple attention mechanisms to enhance multimodal recognition performance. In addition, our attention-based fusion process can acquire comprehensive and diverse features across multiple scales and levels.

### 2.3. Federated Learning

Centralized machine-learning techniques, in which the model is placed on a cloud server or data center and requires large amounts of data to be uploaded from edge nodes, are not scalable for the growing amount of data generated by the IoT [18]. This is particularly problematic for multimodal biometric recognition, which relies on data from various sources including sensors, machines, and other IoT devices. The transmission of raw data also raises concerns around privacy and regulatory compliance, especially since the enacted General Data Protection Regulation (GDPR) [19]. The regulation includes provisions with strict regulations that aim to regulate the use of user data by enterprises.

The FL method of machine learning enables modeling with decentralized data sources and eliminates the need for centralized data, therefore reducing the privacy risks typically associated with traditional centralized machine learning. FL involves creating a global neural network model on a central server that is then downloaded onto the local devices of participating parties. Each party trains the model with their local data and uploads the updated version to the central server. To enhance the security of data transmission and protect sensitive information, this approach adopts the “bringing the code to the data” philosophy [20]. FL has been proposed as a promising technique for a wide range of IoT services, including IoT privacy protection [21,22]. For instance, Zhao et al. [23] proposed a hierarchical crowd-sourcing FL system for IoT data sharing, which collects data from home appliances periodically and trains the machine-learning model, Li et al. [24] developed an FL framework that enables multiple industrial cyber-physical systems to collaboratively create a robust intrusion detection model while ensuring privacy protection. Additionally, Wu et al. [25] demonstrated the effectiveness of FL in the context of IoT-based human activity recognition. We trust that FL may offer significant advantages for IoT multimodal biometric recognition.

## 3. Proposed Model

### 3.1. Feature Extraction Network

In our work, we adopt a strategy of unimodal processing in the early layers of the network, followed by cross-modal fusion in the later layers. To facilitate cross-modal fusion, we first transform the audio and facial features into feature matrices. The reason for this is that lower layers of the network mainly handle low-level features, while higher layers process higher-dimensional features. Low-level features, such as the background noise in audio, do not contain valuable information, making the early fusion of multiple modalities ineffective.

**Figure 1:**
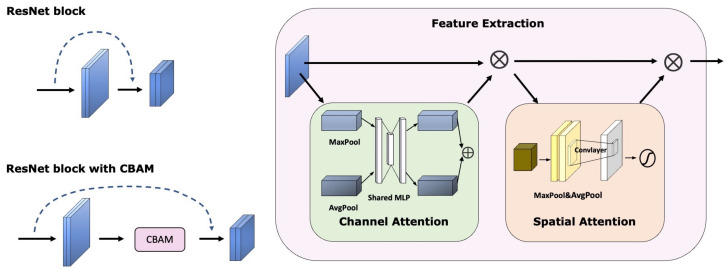
The improved structure of ResNet.

For image input, our feature extraction network is based on the ResNet-34 [26] architecture, we integrated Convolutional Block Attention Module (CBAM) [27] into each residual block of ResNet-34 to combine channel attention and spatial attention. The improved structure is depicted in Figure 1. The CBAM model consists of two distinct sub-modules: the channel attention module (CAM) and the spatial attention module (SAM). The CAM is to identify essential areas in the image with a focus on filtering out irrelevant information. Meanwhile, SAM complements CAM by locating the most significant information after the processing by CAM.

For audio input, while Long Short-Term Memory (LSTM) models are effective in extracting features from sequential data and converting variable-length speech into fixed-length voice features, they may not be suitable for very long speech inputs due to the problem of gradient vanishing [28]. ECAPA-TDNN model [29] introduces several structures such as the Squeeze-and-Excitation (SE) module and Attention Statistics Pooling (ASP) for computing the weight of each frame’s corresponding feature in the speech signal. As a result, the ECAPA-TDNN model has demonstrated remarkable performance in the speaker verification domain.

We propose a modification to the SE module in ECAPA-TDNN that incorporates the advantageous features of the simplified non-local module and the original module, within the global context (GC) modeling [30] framework. This introduces a more effective method for audio input.

**Figure 2:**
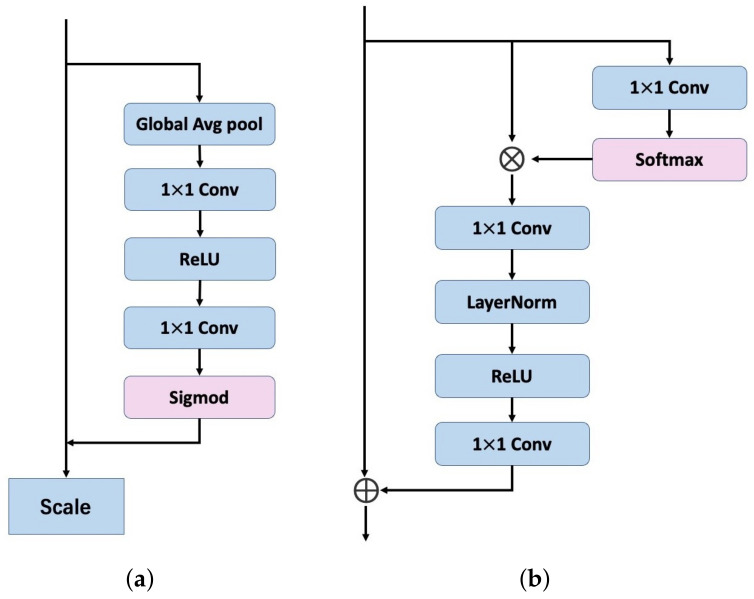
The SE module (**a**) and modified module (**b**).

Figure 2 illustrates the SE module and GC module, the proposed method enables the network to capture both long-range dependencies and local interactions more effectively, therefore increasing its robustness. The final speech feature is obtained using weighted averaging, allowing us to capture speaker factors related to long-term changes with greater accuracy and effectiveness. This is particularly useful in specific corpora where certain frame-level features are more unique and critical in distinguishing speakers than others.

### 3.2. Biometric Modalities Fusion Network

Through the linear layer in the extraction network, the voice clips and face images are transformed into audio embeddings $e_{a}$ and visual embeddings $e_{v}$, we explored two different strategies for the multimodal fusion: simple feature fusion (SFF) and attention-based fusion (AF).

**Figure 3:**
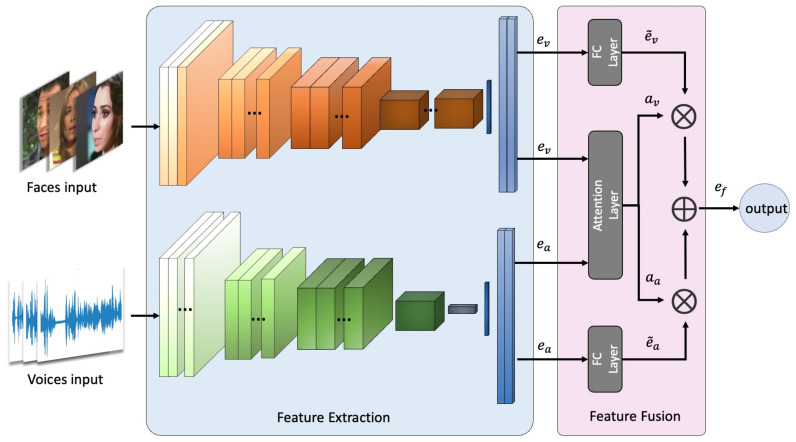
Model architecture of our network with attention-based fusion.

In SFF, we fuse $e_{a}$, $e_{v}$ through vector concatenation, the content vectors from each feature are fused with the same weights, and the fusion embedding is $e_{f}=\left[e_{a},e_{v}\right]$. To increase the performance of multimodal biometric recognition, we implement a soft attention mechanism to fuse features from different modalities, as shown in Figure 3. The fusion network allows us to handle missing or corrupt data from either modality in a natural manner.

First, audio embeddings and face embeddings through attention layers, compute the score as follows:

(1) $${\hat{a}}_{\left\{a,v\right\}}=f_{att}\left(\left[e_{a},e_{v}\right]\right)=W^{T}\left[e_{a},e_{v}\right]+b$$

where weight matrix *W* and bias vector *b* are the learnable parameters of the attention layer.

Through full connection (FC) layers $e_{a}$ and $e_{v}$ are transformed to ${\tilde{e}}_{a}$ and ${\tilde{e}}_{v}$, respectively:

(2) $$\begin{array}{cc} {\tilde{e}}_{a} & =f_{FC\_a}\left(e_{a}\right)\\ {\tilde{e}}_{v} & =f_{FC\_v}\left(e_{v}\right) \end{array}$$

where ${\tilde{e}}_{a}$ and ${\tilde{e}}_{v}$ are better suited for the subsequent fusion process. The fusion embedding $e_{f}$ is then generated through a weighted sum operation:

(3) $$e_{f}=\sum_{i\in \{a,v\}}\alpha_{i}{\tilde{e}}_{i},\;\mathrm{where}\;\alpha_{i}=\frac{\exp\left({\hat{a}}_{i}\right)}{\sum_{k\in \{a,v\}}\exp\left({\hat{a}}_{k}\right)},i\in \left\{a,v\right\}$$

the notation ${\tilde{e}}_{i}$ represents the embeddings that have been projected onto a shared embedding space that is consistent with the linear combination operation.

### 3.3. The Federated AMBR Method

Machine learning has been extensively utilized in IoT for extracting valuable insights from IoT data, therefore facilitating the development of intelligent applications. In the training process of a multimodal biometric recognition network, collecting sufficient and diverse voice and face data is crucial, this task can be challenging due to various factors such as privacy concerns and relevant privacy protection laws and regulations in different countries and regions, such as the GDPR.

Rather than sharing raw IoT data, FL offers an additional approach to distributing learning outcomes while maintaining data privacy. During model training, data owners hailing from various regions possess the capability to leverage their complete set of private data, as opposed to being constrained by the utilization of only partially sensitive data due to privacy apprehensions. Consequently, FL fosters secure data exchange between data owners and data requesters, enhancing the security and reliability of data sharing and ultimately enabling end users to acquire robust network models.

**Figure 4:**
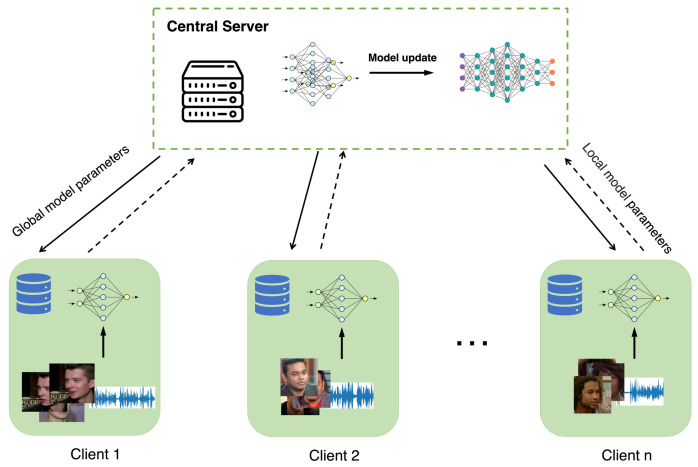
The illustration of our approach.

A multinational company with IoT data in different countries or regions might have different privacy rules to handle data. As shown in Figure 4 we explored a federated approach to train our AMBR network inspired by the Fedavg algorithm [6], enabling collaborative learning between the server and clients from different countries. The detailed process is shown in Algorithm 1, the server is tasked with initializing model parameters, optionally distributing them to clients, collecting the model parameters trained by clients, and performing a weighted average operation. Each client node trains the model using its own local data and then uploads parameters to the server. Models that are trained with different voice and face datasets are averaged by a central server.

In the federated AMBR method, we consider a scenario involving *K* clients, indexed by *k*, and multiple rounds denoted by *r*. At first, the server initializes the global model by requesting the initial parameters $w_{0}$ from a randomly selected client. Within each round, a random subset of *M* clients, referred to as *K*, is selected. The following steps are executed in parallel for each client k∈K. The client *k* executes the client update function, which takes the current model parameters $w_{r}$ as input and returns the updated parameters $w_{r+1}^{k}$. The global model is updated through a weighted averaging of local models generated by the selected clients. The weight assigned to each client is determined by the proportion of its sample size $n_{k}$ to the total data sample size *n*. This weight allocation strategy ensures that clients with larger data volumes have a greater influence on the global model, therefore better reflecting the overall data distribution and incorporating contributions from each client proportionally.

The client update function performed on each client *k* consists of multiple steps. First, the client retrieves the current model parameters *w* from the FL server. Then, for each local epoch *i* ranging from 1 to *E*, the client performs batch-wise computations. Within each batch *b*, the client extracts audio embeddings $e_{a}$ and visual embeddings $e_{v}$ from the local dataset $D_{k}$. These embeddings are subsequently fused using a fusion network. Afterward, the model parameters *w* are fine-tuned and updated using a specific loss function. This results in the creation of a global model that is used for multimodal biometric recognition.
**Algorithm 1** The federated AMBR method. The *K* clients are indexed by *k*, rounds are indexed by *r*, *n* is the number of samples, $\left\{D_{i}\right\}$ represents the individual dataset owned by each client, *E* is the number of local epoch, *B* is the local batch size and *w* is the model parameters**Server executes:** initialize $w_{0}$**for** each round r=1,2,… **do**    K ← (random subset of *M* clients)    **for** each client k∈K **in parallel do**        $w_{r+1}^{k}$← ClientUpdate $\left(k,w_{r}\right)$    **end for**    $w_{r+1}\leftarrow \sum_{k=1}^{K}\frac{n_{k}}{n}w_{t+1}^{k}$**end for** **ClientUpdate**(k,w)**:**//Run on client *k*Get parameters *w* from FL server**for** each local epoch *i* from 1 to *E* **do**    **for** batch b∈B **do**        Extract audio embeddings $e_{a}$ and visual embeddings $e_{v}$ from local dataset $\left\{D_{k}\right\}$        Fuse the $e_{a}$, $e_{v}$ through the fusion network        Fine-tune and update *w* with loss function    **end for****end for**return *w* to Server

## 4. Experiments

### 4.1. Datasets and Training Details

In our study, the VoxCeleb1 and VoxCeleb2 datasets [31,32] were utilized to train and test our AMBR model, which includes large-scale audio-visual data comprising short speech segments extracted from interview videos on YouTube. The training data consists of 1,092,009 video clips extracted from 5994 celebrities in the Voxceleb2 dev sets. The evaluation of the model was conducted using the official trial lists Vox1_O, Vox1_E, and Vox1_H. To guarantee an unbiased evaluation, the validation data do not overlap with the training data.

The original validation set, Vox1_O, contains 37,611 trials with 40 speakers. The extended validation set, Vox1_E, contains 579,818 trials with 1251 speakers. In addition, the hard validation set, Vox1_H, contains a list of 550,894 trials with 1190 speakers, where all speakers are restricted to the same nationality and gender. Using these different sets for evaluation, the performance and generalization of the model could be assessed under varying conditions, including different speaker populations and characteristics.

For facial data, we extracted one image per second, then cropped and scaled them to 224 × 224 pixels to ensure the face remains fully visible, then normalized each pixel value. For audio data, voice clips were extracted from videos and converted into mono, 16-bit samples at a 16 kHz sampling rate, the shorter audio segments were concatenated back-to-back to ensure consistency. To account for various scenarios including background noise and other types of noise, three types of noise from the Musan [33] dataset were added to augment the audio data.

The overall training process involved two stages. During the first stage, we centrally trained the AMBR model and compared it with other biometric recognition systems, each network was trained for 200 epochs with a batch size of 256. We employed the Adam optimizer with an initial learning rate of 0.001. To facilitate the learning process, we applied a learning rate decay of 0.95 every 10 epochs. We optimized the model parameters using the additive angular margin (AAM) SoftMax [34], the AAM-SoftMax loss *L* is defined as:

(4) $$L=-\frac{1}{n}\sum_{i=1}^{n}log\frac{e^{s(cos\left(\theta_{y_{i}}+m\right))}}{e^{s(cos\left(\theta_{y_{i}}+m\right))}+\sum_{j=1,j\neq y_{i}}^{N}e^{s(cos\left(\theta_{j}\right))}}$$

where *N* represents the total number of classes, *n* is the batch size, $\theta_{y_{i}}$ is the angle between the feature embedding of the *i*-th sample and its corresponding class $y_{i}$, hyper-parameter *m* represents the margin, which is used to increase the separation between classes and prevent samples from clustering too closely in the feature space, hyper-parameter *s* is the scaling factor, which adjusts the scale of the angles. The AAM-SoftMax loss function demonstrates unstable convergence when randomly initialized with larger values of *m*. Specifically, the model trained with the widely used configuration, with *m* set to 0.3 and *s* set to 30, exhibits better performance when compared to the vanilla triplet loss [35]. For our experimental setup, the hyper-parameters were set to 0.3 for the *m* and 30 for the *s*. The second stage simulated the FL scenario by partitioning the dataset among five clients under three distinct settings, training data were distributed among all clients.

### 4.2. Unimodal Biometric Recognition

The Equal Error Rate (EER) is a critical indicator for evaluating the reliability of the biometric recognition system, which is determined by the intersection point of the False Acceptance Rate (FAR) and False Rejection Rate (FRR). The FAR and FRR can be calculated as follows:

(5) $$\mathrm{FAR}=\frac{\mathrm{FP}}{\mathrm{FP}+\mathrm{TN}}$$

(6) $$\mathrm{FRR}=\frac{\mathrm{FN}}{\mathrm{TP}+\mathrm{FN}}$$

where FP stands for false positives, which represents the number of actual negatives classified as positive. Similarly, TN refers to true negatives, TP means true positive, and FN means false negatives. A lower EER value implies higher reliability and accuracy of the biometric recognition system.

First, we trained two separate unimodal biometric recognition networks to test the performance of our feature extraction network, the comparisons of EER with baseline techniques and the proposed method are provided for Vox1_O, E, and H trails. Table 2 shows the results of the unimodal person recognition experiment on the trail sets.

After analyzing our results, we observed that although the inclusion of MUSAN augmented audio features led to an enhancement in recognition accuracy, the performance of the audio system was significantly inferior to that of the visual system. We also discovered that the attention-based model surpassed the baseline model, with the superior performance being attributed to the ability of attention mechanisms to model the global features of faces and voices, making it more suitable for feature extraction.

We also compared our feature extraction network with several popular networks including VGG-M [36], VGG-16 [37], ResNet-18, and ResNet-34. To ensure a fair comparison, we augmented audio clips with MUSAN in all networks. On the Vox1_E test set, the comparison of VGG-M shows that the EER of voice recognition improved by 15%, and the EER of face recognition led to a relative improvement of 7.6% compared to ResNet-34. Table 3 demonstrates that our proposed method is comparable to other unimodal systems and the improved modules can help the network develop a better ability to capture relevant information for recognition.

### 4.3. Multimodal Biometric Recognition

The fusion network utilizes face and voice embeddings extracted from the feature extraction networks described in Section 4.2. Table 4 presents a comparative analysis of multimodal and unimodal biometric recognition. The results suggest that employing SFF can enhance performance. Moreover, the AF method we proposed outperformed the SFF method. The AF method achieved remarkable performance with 0.68%, 0.47%, and 0.80% EER on trial Vox1_O, Vox1_E, and Vox1_H, respectively, these results indicated a significant improvement over the SFF method, with improvements of 27%, 20%, and 23% for the corresponding validation sets. SFF was found to be inadequate in effectively utilizing multiple types of features, as the content vectors from each feature were fused using the same weights. In contrast, the attention-based fusion model effectively modulated the correlation between the face and voice features by calculating the fusion embedding using a weighted sum. Compared with other multimodal recognition systems, the proposed method exhibited EER improvements of 24%, 9.3%, and 13% over their respective method on the trail lists Vox1_O. Therefore, our proposed system effectively extracts multimodal biometric information to accurately determine identity.

### 4.4. The Experimental Results in FL Settings

To evaluate the AMBR with FL approach, we conducted experiments on the VoxCeleb1 dataset. Specifically, we selected 50 speakers from India, the USA, Canada, the UK, and Austria, resulting in 7500 face-voice pairs. We divided these pairs into 6000 pairs of training data and 1500 pairs of test data. We created two additional datasets by subsampling 50% and 30% of the original training data while leaving the test data unchanged. We denoted the aforementioned three experimental data configurations as Setting 1, Setting 2, and Setting 3, correspondingly. By utilizing subsampling datasets, we aimed to better evaluate the performance differences between our proposed approach and other methods under different scenarios.

In our experiments, we utilized a central server and five client devices, each client was restricted to speakers of a single nationality to mimic the IoT scenario, where participants utilize their own biometric data to train the model without compromising each other’s private information. For a fair comparison, we set the communication round, local epoch, and local batch size to 200, 5, and 10, respectively. We maintained a learning rate of 0.01 in the centralized standard SGD within the FL framework.

**Figure 5:**
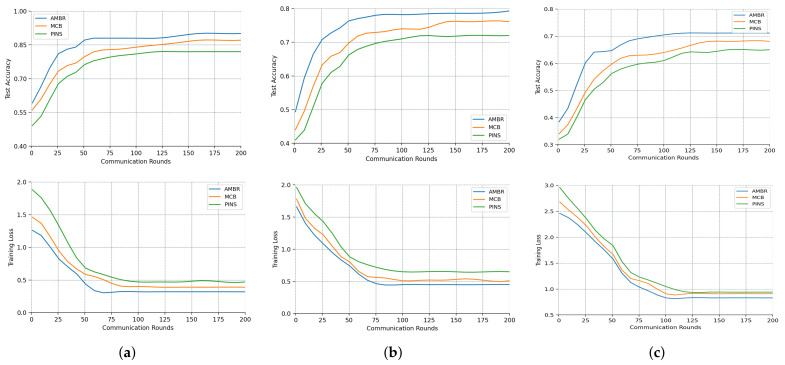
Evaluate different methods for accuracy and loss under different settings. (**a**) Setting 1; (**b**) Setting 2; (**c**) Setting 3.

Compared with other methods, our proposed method demonstrated superior accuracy and convergence speed as revealed by Figure 5. The reduced datasets lack the full diversity and richness of the original data, resulting in lower performance. A larger training set provides more samples for model training, improving generalization ability and performance, and facilitates a more comprehensive representation of the data, reducing bias and enhancing the model’s understanding of data distribution and features. We also trained a centralized model on the entire dataset, which achieved an accuracy of 0.974. We observed that despite the performance gap of the FL model, our proposed method outperformed other methods, confirming the effectiveness of our approach.

Specifically, our approach outperformed method MCB by 8%, 7.3%, and 6% in the three distinct settings, and it showed 3.9%, 3.2%, and 2.7% higher accuracy than method PINS. The superiority of our proposed method becomes more pronounced as the experimental data volume increases. The decreasing trend in loss values across different settings indicates that our AMBR method demonstrates faster convergence compared with other methods. The decreasing trend in loss values across different settings confirmed that our proposed method exhibits faster convergence compared with the other methods. In FL applications, this accelerated convergence speed can significantly reduce the communication bandwidth pressure. Consequently, our method achieves efficient utilization of communication bandwidth while ensuring optimal model performance.

## 5. Discussion

In this work, we developed a multimodal biometric recognition network, AMBR, which utilizes attention mechanisms to selectively attend to the most relevant modality of the inputs to generate a powerful fusion representation that is suitable for the biometric recognition task in IoT. The effectiveness of two feature fusion strategies, simple feature fusion and attention-based fusion were compared, and the results indicated that attention-based fusion is more efficient in combining the biometric features. Our proposed AMBR network achieved EER results of 0.68%, 0.47%, and 0.80% on the Vox1_O, Vox1_E, and Vox1_H test sets, showing that our AMBR outperforms the traditional unimodal systems significantly.

Furthermore, we proposed the utilization of FL to safeguard user data privacy during the training process, which enables individual IoT clients to collaborate in training the central model while ensuring the confidentiality of sensitive user data by preventing raw data from leaving devices. Our experiments showed that the proposed approach can effectively train with privacy preservation in place.

In future research, it is crucial to explore improved FL aggregation methods that can lead to enhanced performance gains. In addition, extending our experiments to include a larger number of clients and diverse data distributions will enable us to investigate the trade-off between preserving privacy and achieving high model accuracy. Moreover, research on lightweight multimodal networks is also a promising direction. By training a lightweight model within the context of FL, we may potentially achieve even greater performance improvements and reduce communication overhead, as the model’s structure and parameter count can significantly influence these factors.

## Figures and Tables

**Table 1:** The comparison of existing multimodal fusion methods.

Model	Architecture	Features	Fusion Strategy
Luo et al. [8]	CNN + RNN	voice and text	Fuse the audio and handcrafted low-level descriptor through simple vector concatenation.
Micucci et al. [9]	CNN	palmprint and hand-geometry	Score level fusion, sum the weighted scores from each modality.
Sell et al. [10]	DNN + CNN	face and voice	Converting the output scores generated from unimodal verification systems into log-likelihood ratios.
PINS [11]	VGG-M	face and voice	Establish a joint embedding between faces and voices.
EmoRL-Net [12]	ResNet-18	face and voice	Project the representation of two full connection layers into a spherical space.

**Table 2:** The comparison of unimodal recognition networks with the baseline.

Test Modality	Method	EER(%)		
		Vox1_O	Vox1_E	Vox1_H
Audio	ECAPA-TDNN	1.86	1.98	3.16
Audio	ECAPA-TDNN *	1.82	1.93	3.02
Audio	AMBR	1.72	1.76	2.80
Audio	AMBR *	1.65	1.73	2.71
Visual	ResNet-34	1.61	1.45	2.01
Visual	AMBR	1.47	1.34	1.68

Method with * used MUSAN to augment audio.

**Table 3:** Unimodal person recognition networks EER (%) comparison.

Test Modality	Method	EER(%)		
		Vox1_O	Vox1_E	Vox1_H
Audio	ResNet-18	2.35	2.42	3.67
Audio	ResNet-34	2.01	2.10	3.24
Audio	VGG-M	1.96	2.04	3.26
Audio	AMBR	1.65	1.73	2.71
Visual	ResNet-18	1.74	1.66	2.08
Visual	VGG-16	1.80	1.71	2.15
Visual	AMBR	1.47	1.34	1.68

**Table 4:** The EER (%) of multimodal biometric recognition methods.

Test Modality	Method	EER(%)		
		Vox1_O	Vox1_E	Vox1_H
Audio	AMBR	1.65	1.73	2.71
Visual	AMBR	1.47	1.34	1.68
Visual + Audio	Sari et al. [38]	0.90	-	-
Visual + Audio	MCB [39]	0.75	0.68	1.13
Visual + Audio	PINS [11]	0.79	0.50	0.91
Visual + Audio	AMBR with SFF	0.93	0.59	1.04
Visual + Audio	AMBR with AF	0.68	0.47	0.80

## Data Availability

The datasets used during this study are available at the URL: https://www.robots.ox.ac.uk/~vgg/data/voxceleb (accessed on 24 February 2023).
